# Supplementary material for: Short-Term Adverse Events and Antibody Response to the BNT162b2 SARS-CoV-2 Vaccine in 4156 Health Care Professionals
Source: Vaccines (Basel). 2022 Mar 13;10(3):439. doi: 10.3390/vaccines10030439 (PMC8950377; doi:10.3390/vaccines10030439)
Supplement: Supplementary file 1 [file vaccines-10-00439-s001.zip › vaccines-1593302-supplementary.pdf]

Supplementary Table S1. Type and duration of self-reported adverse events. Continuous variables are expressed as median (IQR).

	Total		Dose 1		Dose 2	
**Adverse event**	**N (%)**	**Duration (days)**	**N (%)**	**Duration (days)**	**N (%)**	**Duration (days)**
Local reaction	1858 (30.4%)	3 (1-3)	905 (28.6%)	3 (1-3)	953 (32.3%)	3 (1-3)
Asthenia	1444 (23.6%)	3 (1-3)	545 (17.3%)	1 (1-3)	899 (30.4%)	3 (1-3)
Headache	1033 (16.9%)	1 (1-3)	390 (12.3%)	1 (1-3)	643 (21.8%)	1 (1-3)
Muscle pain	959 (15.7%)	3 (1-3)	294 (9.3%)	3 (1-3)	665 (22.5%)	3 (1-3)
Joint pain	708 (11.6%)	3 (1-3)	183 (5.8%)	3 (1-3)	525 (17.8%)	3 (1-3)
Fever	539 (8.8%)	1 (1-3)	81 (2.6%)	1 (1-3)	458 (15.5%)	1 (1-3)
>38°	251 (46.6%)	1 (1-3)	23 (28.4%)	1 (1-3)	228 (49.8%)	1 (1-3)
Lymph node enlargement	269 (4.4%)	3 (3-7)	65 (2.1%)	3 (3-7)	204 (6.9%)	3 (3-7)
Chills	685 (11.2%)	1 (1-3)	181 (5.7%)	1 (1-3)	504 (17.1%)	1 (1-3)
Rash	93 (1.5%)	3 (1-7)	45 (1.4%)	3 (3-7)	48 (1.6%)	3 (1-7)
Diffuse	18 (19.4%)	3 (1-7)	8 (17.8%)	7 (1-7)	10 (20.8%)	2 (1-7)
Anxiety	43 (0.7%)	3 (1-7)	21 (0.7%)	1 (1-3)	22 (0.8%)	3 (1-7)
Presyncope	115 (1.9%)	1 (1-3)	44 (1.4%)	1 (1-3)	71 (2.4%)	1 (1-3)
Syncope	8 (0.1%)	2 (1-3)	1 (0.03%)	3	7 (0.2%)	1 (1-3)
Abdominal pain	103 (1.7%)	3 (1-3)	29 (0.9%)	3 (1-7)	74 (2.5%)	3 (1-3)
Insomnia	191 (3.1%)	1 (1-3)	56 (1.8%)	1 (1-5)	135 (4.6%)	1 (1-3)
Diarrhea	132 (2.2%)	3 (1-3)	43 (1.6%)	3 (1-3)	89 (3.0%)	1 (1-3)
Nausea	296 (4.8%)	1 (1-3)	90 (2.9%)	1 (1-3)	206 (7.0%)	1 (1-3)
Vomiting	49 (0.8%)	1 (1-3)	14 (0.4%)	1 (1-3)	35 (1.2%)	1 (1-1)
Angioedema	28 (0.5%)	3 (1-7)	9 (0.3%)	1 (1-3)	19 (0.6%)	3 (1-7)
Transient facial palsy	12 (0.2%)	1 (1-1)	6 (0.2%)	1 (1-2)	6 (0.2%)	1 (1-3)
Hypotension	35 (0.6%)	1 (1-3)	13 (0.4%)	1 (1-1)	22 (0.8%)	1 (1-3)
Sweating	193 (3.2%)	1 (1-3)	59 (1.9%)	1 (1-3)	134 (4.5%)	1 (1-3)
Tachycardia	117 (1.9%)	1 (1-3)	38 (1.2%)	1 (1-3)	79 (2.7%)	1 (1-3)
Chest pain	56 (0.9%)	3 (1-3)	19 (0.6%)	3 (1-3)	37 (1.3%)	3 (1-3)

Dyspnea	47 (0.8%)	3 (1-7)	17 (0.5%)	3 (1-14)	30 (1.0%)	3 (1-7)
Any other	76 (1.2%)	1 (1-7)	27 (0.9%)	1 (1-7)	49 (1.7%)	1 (1-7)
